# Against the stream: Antidepressants are not antidepressants – an alternative approach to drug action and implications for the use of antidepressants

**DOI:** 10.1192/bjb.2017.11

**Published:** 2018-02

**Authors:** Joanna Moncrieff

**Affiliations:** 1 University College London, UK

## Abstract

Although antidepressants are regarded as effective and specific treatments, they are barely superior to placebo in randomised trials, and differences are unlikely to be clinically relevant. The conventional disease-centred understanding of drug action regards antidepressants as targeting an underlying brain process, but an alternative ‘drug-centred’ view suggests they are psychoactive substances that modify normal mental states and behaviour. These alterations, such as numbing of emotions, may reduce feelings of depression, and also create amplified placebo effects in randomised trials. Patients should be informed that there is no evidence that antidepressants work by correcting a chemical imbalance, that antidepressants have mind-altering effects, and that evidence suggests they produce no noticeable benefit compared with placebo.

**Declaration of interest:**

The author is co-chairperson of the Critical Psychiatry Network.

Antidepressants are by far the most commonly prescribed class of drug for mental disorders, and their use continues to rise.^1^ Huge marketing campaigns have persuaded the general public that depression is a ‘chemical imbalance’ that antidepressants can help reverse. Professional organisations claim that antidepressants are an effective and specific treatment for depression, and that they are considerably more effective than placebo. The Royal College of Psychiatrists' current information leaflet suggests that 50–65% of people who are given an antidepressant will show ‘much improvement’ within 3 months, compared with only 25–30% on placebo.^2^

## The evidence base

Overall, randomised controlled trials show that depression ratings decrease slightly more with antidepressants than placebo. Studies are inconsistent, however, and differences are small, especially when unpublished trials are included. Reviews of the literature on tricyclic and older antidepressants concluded that ‘in well-designed studies, the differences between antidepressants and placebo are not impressive’.^3^ Meta-analyses of trials of selective serotonin reuptake inhibitors (SSRIs) and other modern antidepressants that include unpublished trials have found mean differences between antidepressants and placebo ranging from 1.80 to 2.55 points on the widely used Hamilton Rating Scale for Depression.^4^^–^^6^

The clinical significance of such small differences is doubtful. The Hamilton scale has a total score of 54 points. A recent analysis comparing scores on the Hamilton scale with scores on the observer-rated Clinical Global Impression (CGI) scale suggests that a difference of three points on the Hamilton scale is equivalent to a rating of ‘no difference’ on the CGI scale, while a difference of eight points is required to obtain CGI scale ratings of ‘mild improvement’.^7^^,^^8^ Antidepressant/placebo differences therefore appear to fall well below levels required to make a noticeable difference in someone's condition.

## Antidepressant effects and severity

It is often suggested that antidepressants are more effective, or perhaps only effective, in severe depression, and that this can explain their poor performance relative to placebo in trials with mixed populations. Some meta-analyses have found a gradient between the size of the antidepressant/placebo difference and the severity of initial depression across trials,^5^^,^^9^ although differences in people with the most severe depression still fall well below those equating to ‘mild improvement’ on the CGI. Other meta-analyses have not identified a severity gradient.^10^^,^^11^

Older evidence suggests that antidepressants are not particularly helpful for inpatients with depression. A Medical Research Council trial, for example, found little difference between imipramine, phenelzine and placebo.^12^ Trials of antidepressants for the treatment of depression in people with bipolar disorder have also found no differences between antidepressants and placebo.^13^

## Antidepressant effects and the drug-centred model of drug action

The accepted view of drug action in psychiatry is that psychiatric drugs work by targeting a putative underlying brain abnormality. I have called this the ‘disease-centred’ model of drug action. An alternative explanation is the ‘drug-centred’ model, which suggests that psychiatric drugs influence symptoms of mental disorder and distress through their psychoactive effects. ‘Psychoactive’ drugs, sometimes referred to as ‘mind-altering drugs’, include recreational drugs, drugs prescribed for mental health problems and some other medical drugs (e.g. steroids, anticholinergics). They modify normal thoughts, emotions and behaviours in characteristic ways. According to the drug-centred model of drug action, for example, antipsychotics reduce the symptoms of psychosis through their ability to produce a state of mental slowing and emotional restriction, a state they produce in animals and humans, regardless of the presence of psychiatric or behavioural problems. Anxiolytics reduce symptoms of anxiety through their well-known sedative and relaxant effects, which occur independently of any psychiatric disorder.

Elsewhere, I have outlined the lack of evidence for the disease-centred view of drug action for any class of psychiatric medication.^14^ The serotonin and noradrenaline theories of depression, which appear to explain the action of antidepressants in a disease-centred manner, are not supported by evidence or expert opinion.^15^^,^^16^ Moreover, numerous randomised trials have shown that drugs that are not considered as antidepressants, and have actions on other neurotransmitter systems, including benzodiazepines, opiates, stimulants and antipsychotics, are as effective as recognised antidepressants in people with depression.^15^

The drug-centred model suggests that the mental and physical alterations produced by antidepressants account for the differences between antidepressants and placebo in randomised trials. The psychoactive effects of individual antidepressants vary in strength and character, depending on chemical class and composition. Tricyclic drugs are strongly sedating and impair psychological test performance.^17^ SSRIs have weaker and more subtle effects, but can induce a state of emotional numbing or restriction, lethargy, reduced libido and sexual impairment. They also occasionally produce a state of agitation and tension, especially in young people.^18^ Antidepressant-induced emotional numbness may directly reduce the intensity of people's feelings, but mental and physical alterations may also produce an amplified placebo effect, by revealing to people participating in randomised trials that they are taking an active drug. The fact that drug/placebo differences are so small, however, suggests that antidepressant-induced alterations are not clinically useful, whether they act through pharmacological or psychological means.

## Adverse effects

By emphasising that psychiatric drugs change the normal state of the brain and body, the drug-centred model highlights the likelihood of adverse effects. Although modern antidepressants are usually well-tolerated, there is mounting evidence of less common but serious effects, including increased suicidal thoughts,^19^ fetal malformations,^20^ bleeding, a prolonged and severe withdrawal syndrome,^21^ and persistent sexual dysfunction after discontinuation.^22^ The widespread use of antidepressants may also produce nocebo effects by undermining people's sense of self-efficacy, potentially setting them up for a lifetime of chronicity and dependence on services.

## Conclusions

The public have been led to believe that depression is caused by a chemical imbalance that antidepressants help to rectify; however, there is no current evidence that any sort of drug specifically targets an underlying biological abnormality, and whether there is an underlying brain state or states specific to the experience of depression has not been demonstrated. Amplified placebo effects and the subtle emotional alterations produced by antidepressants may account for the small differences between antidepressants and placebo found in some randomised controlled trials, but these small differences are unlikely to translate into a clinically meaningful effect. Doctors need to share this evidence with patients who are considering taking an antidepressant. Doctors should also help people to consider the pros and cons of using a mind-altering drug, such as an antidepressant, in relation to each individual's particular situation. This should include discussion of alternative ways of achieving desired outcomes, using strategies that do not carry the inherent risks of drug treatment.

Although the discovery of a specific antidepressant agent in the future cannot be ruled out, it is possible that we misunderstand the nature of depression, and that regarding it as a discrete and universal disorder may have raised false hopes about the chance of a generally applicable ‘cure’ or treatment. The alternative view of depression as part of the spectrum of meaningful human responses to the world suggests that drugs will only dull the experience. In the end, the situation that provoked the negative emotion needs to be addressed. Depression is a signal that change is needed in some aspect of life.

## About the author

**Joanna Moncrieff** is a Reader in Critical and Social Psychiatry at University College London, and an honorary Consultant Psychiatrist at North East London Foundation Trust. She is also a founder and co-chair person of the Critical Psychiatry Network.
